# Supplementary material for: Impact of health systems interventions in primary health settings on type 2 diabetes care and health outcomes among adults in West Africa: A systematic review
Source: PLoS One. 2025 Apr 8;20(4):e0319478. doi: 10.1371/journal.pone.0319478 (PMC11978094; doi:10.1371/journal.pone.0319478)
Supplement: S2 File — (PDF) [file pone.0319478.s003.pdf]

Database	Search Query	Total number of articles
PubMed	(((((("Health Policy"[MeSH Terms] OR "National Health Programs"[MeSH Terms] OR "health system interventions"[All Fields] OR "Health Services"[MeSH Terms] OR "Delivery of Health Care"[MeSH Terms] OR "Healthcare Financing"[MeSH Terms] OR "insurance, health, reimbursement"[MeSH Terms] OR "Health Information Systems"[MeSH Terms] OR "Equipment and Supplies"[MeSH Terms] OR "Task Shifting"[MeSH Terms] OR "Health Personnel"[MeSH Terms] OR "Health Services"[All Fields] OR "healthcare delivery"[All Fields] OR "delivery of healthcare"[All Fields] OR "health workers"[All Fields]) AND "Primary Health Care"[MeSH Terms]) OR "Health Facilities"[MeSH Terms]) AND "diabetes mellitus, type 2"[MeSH Terms]) OR "Type 2 diabetes"[All Fields] OR "glycemic control"[All Fields] OR "Diabetes Complications"[MeSH Terms] OR "hyperglycemia"[All Fields]) AND ("africa, western"[MeSH Terms] OR "Benin"[All Fields] OR "Burkina Faso"[All Fields] OR "Cabo Verde"[All Fields] OR "Cote d'ivoire"[All Fields] OR "Gambia"[All Fields] OR "GHANA"[All Fields] OR "Guinea"[All Fields] OR "Guinea-Bissau"[All Fields] OR "Liberia"[All Fields] OR "Mali"[All Fields] OR "Mauritania"[All Fields] OR "Niger"[All Fields] OR "Nigeria"[All Fields] OR "Senegal"[All Fields] OR "Sierra Leone"[All Fields] OR "Togo"[All Fields] OR "West Africa"[All Fields])) AND (2000:2024[pdat])	2,098
Google Scholar	“Health system interventions” OR “health programmes” OR “health policy” OR “screening” AND “type 2 diabetes” AND West Africa	898
CINAHL	health facility OR primary healthcare OR health services OR delivery of health care OR Health Care Facilities, Manpower, and Services OR healthcare financing OR Insurance, Health, Reimbursement OR health information systems OR equipment and supplies AND type 2 diabetes OR glycemic control OR hyperglycemia OR diabetes mellitus type 2 OR diabetes complications OR diabetes adherence AND Benin OR burkina faso OR cabo verde OR cote d'ivoire OR gambia OR ghana OR guinea OR guinea bissau OR liberia OR mali OR mauritania OR niger OR nigeria OR senegal OR sierra leone OR togo AND rct or randomised control trial OR clinical controlled trial	328
Cairn. Info	1. 'accès' ET 'diabètes' ET 'résultats' ET 'afrique de l'ouest'<br>2. interventions pour le diabète du type 2 en Afrique de l'ouest 3. interventions pour les diabetes en Afrique de l’ouest<br>4. diabète de type ii en afrique de l'ouest<br>5. diabète ET 'West Africa'<br>6. ‘diabetes’ and ‘africa’<br>7. 'diabetes' AND 'west africa'<br>8. 'type 2 diabetes' and 'west africa'<br>9. 'interventions' and 'type 2 diabetes' and 'west africa'<br>10. 'interventions' and 'type 2 diabetes'<br>11. 'interventions' and 'type 2 diabetes' and 'outcomes'<br><br>1 OR 2 OR 3 OR 4 OR 5 OR 6 OR 7 OR 8 OR 9 OR 10 OR 11	23
