# Supplementary material for: Impact of health systems interventions in primary health settings on type 2 diabetes care and health outcomes among adults in West Africa: A systematic review
Source: PLoS One. 2025 Apr 8;20(4):e0319478. doi: 10.1371/journal.pone.0319478 (PMC11978094; doi:10.1371/journal.pone.0319478)
Supplement: S3 File — (PDF) [file pone.0319478.s004.pdf]

Cochrane Risk of Bias Tool

Type of Bias	Description	High Risk of Bias	Low Risk of Bias	Unclear Risk of Bias	Reviewer Assessment	Reviewer Comments
Selection bias<br>***Random sequence generation***	Described the method used to generate the allocation sequence in sufficient detail to allow an assessment of whether it should produce comparable groups	Selection bias (biased allocation to interventions) due to inadequate generation of a randomized sequence	Random sequence generation method should produce comparable groups	Not described in sufficient detail	**High**<br>**Low**<br>**Unclear**	
Selection bias<br>***Allocation concealment***	Described the method used to conceal the allocation sequence in sufficient detail to determine whether intervention allocations could have been foreseen before or during enrollment	Selection bias (biased allocation to interventions) due to inadequate concealment of allocations prior to assignment	Intervention allocations likely could not have been foreseen in before or during enrollment	Not described in sufficient detail	**High**<br>**Low**<br>**Unclear**	
Reporting bias<br>***Selective reporting***	Stated how the possibility of selective outcome reporting was examined by the authors and what was found	Reporting bias due to selective outcome reporting	Selective outcome reporting bias not detected	Insufficient information to permit judgment†	**High**<br>**Low**<br>**Unclear**	
Other bias<br>***Other sources of bias***	Any important concerns about bias not addressed above*	Bias due to problems not covered elsewhere in the table	No other bias detected	There may be a risk of bias, but there is either insufficient information to assess whether an important risk of bias exists or insufficient rationale or evidence that an identified problem will introduce bias	**High**<br>**Low**<br>**Unclear**	
Performance bias<br>***Blinding (participants and personnel)***	Described all measures used, if any, to blind study participants and personnel from knowledge of which intervention a participant received. Provided any information relating to whether the intended blinding was effective.	Performance bias due to knowledge of the allocated interventions by participants and personnel during the study.	Blinding was likely effective.	Not described in sufficient detail	**High**<br>**Low**<br>**Unclear**	
Detection bias<br>***Blinding (outcome assessment)***	Described all measures used, if any, to blind outcome assessors from knowledge of which intervention a participant received. Provided any information relating to whether the intended blinding was effective.	Detection bias due to knowledge of the allocated interventions by outcome assessors.	Blinding was likely effective.	Not described in sufficient detail	**High**<br>**Low**<br>**Unclear**	
Attrition bias<br>***Incomplete outcome data***	Described the completeness of outcome data for each main outcome, including attrition and exclusions from the analysis. Stated whether attrition and exclusions were reported, the numbers in each intervention group (compared with total randomized participants), reasons for attrition/exclusions where reported.	Attrition bias due to amount, nature or handling of incomplete outcome data.	Handling of incomplete outcome data was complete and unlikely to have produced bias	Insufficient reporting of attrition/exclusions to permit judgment (e.g., number randomized not stated, no reasons for missing data provided)	**High**<br>**Low**<br>**Unclear**	

JBI Critical Appraisal Checklist for Quasi-Experimental Studies (non-randomized experimental studies)

Reviewer: _____________ Date: _______________ Author: _________________ Year: ________________

	**High**	**Low**	**Unclear**	**Not applicable**
Is it clear in the study what is the 'cause' and what is the ‘effect’ (i.e. there is no confusion about which variable comes first)?				
Were the participants included in any comparisons similar?				
Were the participants included in any comparisons receiving similar treatment/care, other than the exposure or intervention of interest?				
Were there multiple measurements of the outcome both pre and post the intervention/exposure?				
Was follow up complete and if not, were differences between groups in terms of their follow up adequately described and analyzed?				
Were the outcomes of participants included in any comparisons measured in the same way?				
Were outcomes measured in a reliable way?				
Was appropriate statistical analysis used?				

Tool for assessing risk of bias for observational studies

**Type of bias**	**Study design**		
	**Cross sectional**	**Case control**	**Cohort**
Selection bias	Was the study population selected appropriate?		
	Was the sample representative of its target population?	Were the controls randomly selected from the same population as the cases?	Was an appropriate control group used?<br>Was follow up sufficiently complete?
Differential misclassification	Did the assessment of the exposure or outcome differ according to the patient status?	Did the exposure assessment differ for cases and controls?	Did the outcome assessment differ for exposed and non-exposed?
Non-differential misclassification	Were valid methods used for measuring hypertension awareness, treatment or control and medication adherence?		
Confounding	Was any strategy undertaken to control for potential confounders?<br>1. At the design stage (restriction, matching)<br>2. At the analysis stage (stratification, multivariable analysis)		

Define each domain as low risk of bias, unclear risk of bias or high risk of bias
